# Effectiveness of the 5A Self‐Management Nursing Intervention on Psychosocial Adaptation Among Patients With Diabetes: A Randomized Controlled Trial

**DOI:** 10.1155/nrp/2814645

**Published:** 2026-05-18

**Authors:** Rafat Rezapour-Nasrabad, Sina Nasrollahi-Nasrabad, Sohaila Dehno Khalaji, Seyedeh Narjes Mousavizadeh

**Affiliations:** ^1^ Department of Psychiatric Nursing and Management, School of Nursing and Midwifery, Shahid Beheshti University of Medical Sciences, Tehran, Iran, sbmu.ac.ir; ^2^ Department of Medicine and Surgery, University of Parma, Via Volturno 39, Parma, 43125, Italy, unipr.it; ^3^ Student Research Committee, School of Nursing and Midwifery, Shahid Beheshti University of Medical Sciences, Tehran, Iran, sbmu.ac.ir

**Keywords:** 5A model, educational intervention, psychosocial adaptation, self-management, type 2 diabetes

## Abstract

**Background:**

Diabetes management requires not only biomedical control but also psychosocial adaptation, which is often overlooked in routine care. The 5A model (Assess, Advise, Agree, Assist, and Arrange) has shown promise in chronic disease management, yet its impact on psychosocial outcomes in diabetes remains underexplored.

**Aim:**

This study aimed to determine the effect of the 5A self‐management program on psychosocial adaptation among hospitalized patients with type 2 diabetes in selected teaching hospitals affiliated with Shahid Beheshti University of Medical Sciences.

**Methods:**

In this randomized controlled trial (RCT), 80 patients with type 2 diabetes who met the inclusion criteria were selected through convenience sampling and randomly assigned to either the intervention group (*n* = 40) or the control group (*n* = 40). The intervention group received the 5A self‐management program, while the control group received routine care. Data were collected using demographic questionnaires, the Diabetes Knowledge Questionnaire, the Diabetes Self‐Management Questionnaire, and the Derogatis Psychosocial Adjustment Questionnaire. Data analysis was performed using SPSS Version 20 with descriptive and inferential statistical tests.

**Results:**

The findings showed no significant differences between the two groups in demographic characteristics (*p* > 0.05). After the intervention, the mean score of psychosocial adaptation in the intervention group (58.6 ± 7.9) was significantly lower than that in the control group (72.4 ± 8.3) (*p* < 0.001). Within‐group comparison revealed a significant decrease in PAIS scores (indicating improvement) in the intervention group.

**Conclusion:**

The 5A self‐management program can be considered an effective intervention for improving psychosocial adaptation among patients with type 2 diabetes. It is recommended that this program be utilized in the healthcare system to enhance psychosocial adaptation in these patients.

## 1. Introduction

Diabetes is one of the most common chronic diseases and a major global health challenge, described by the World Health Organization as a “silent epidemic” [1]. It is characterized by chronic hyperglycemia and can lead to numerous complications, including cardiovascular disease, nephropathy, retinopathy, and neuropathy [2]. In addition to physical complications, diabetes negatively impacts the psychosocial aspects of patients’ lives, including anxiety, depression, stress, and reduced quality of life [3, 4].

According to the International Diabetes Federation, approximately 536 million people worldwide were living with diabetes in 2021, and this number is projected to rise to 783 million by 2045 [5]. In Iran, the prevalence of diabetes has been reported to be around 11%–12% [6–8]. This continuous rise in prevalence imposes a considerable burden on healthcare systems and national economies [9, 10].

Psychosocial adaptation refers to patients’ capacity to adjust their lives to the disease. It includes the ability to control or minimize the impact of the illness on physical, psychological, and social well‐being [11]. Patients with diabetes face multiple challenges that can affect their adaptation, such as the need for lifestyle modification, adherence to dietary restrictions, regular physical activity, and medication use [12, 13].

One of the main strategies for managing the disease and preventing diabetes‐related complications is the implementation of self‐management programs [14, 15]. Self‐management is defined as an individual’s ability to manage symptoms, treatment, and the physical, psychological, and social consequences of living with a chronic condition [16–18]. The 5A self‐management model, developed by Glasgow and colleagues in 2003, is an evidence‐based approach designed to promote behaviors change and facilitate self‐management in patients with chronic diseases [19, 20]. This model includes five stages: Assess, Advise, Agree, Assist, and Arrange [19, 21].

In the Iranian healthcare system, despite the high prevalence of diabetes, routine care is often limited to clinical assessments and prescriptive medical advice, frequently overlooking the psychosocial and behavioral dimensions of the disease [22, 23]. There is a significant gap in structured, longitudinal support for patients, particularly in busy teaching hospitals where time constraints prevent healthcare providers from engaging in collaborative goal‐setting. The 5A model is specifically relevant in this context as it provides a systematic, step‐by‐step framework to bridge this gap. By moving beyond traditional didactic education and focusing on personalized barriers and social support, the 5A framework can address the unmet psychosocial needs of Iranian patients, potentially improving long‐term adherence and adaptation within the existing healthcare infrastructure.

Given the high prevalence of diabetes in Iran and worldwide, its adverse effects on patients’ psychosocial adaptation, and the importance of self‐management in disease control, the present study was conducted to determine the effect of the 5A self‐management program on psychosocial adaptation among hospitalized patients with type 2 diabetes in selected teaching hospitals affiliated with Shahid Beheshti University of Medical Sciences (SBUMS).

## 2. Methods

### 2.1. Study Design and Population

This study was a randomized controlled trial (RCT) conducted in 2024 among hospitalized patients with type 2 diabetes. Contrary to a single‐center design, this study was conducted across five distinct teaching hospitals affiliated with SBUMS. The rationale for selecting these specific centers (Shohada Tajrish, Ayatollah Taleghani, Shahid Modarres, Imam Hossein, and Labbafinejad) was to enhance the study’s generalizability and capture a more diverse patient demographic, while ensuring that the baseline standard of care remained consistent across all sites. A total of 46 patients were recruited from Shohada Tajrish, Ayatollah Taleghani, and Shahid Modarres hospitals (19 in the intervention group and 27 in the control group), and 34 patients were recruited from Imam Hossein and Labbafinejad hospitals (21 in the intervention group and 13 in the control group).

### 2.2. Sampling and Sample Size

Using the sample size calculation formula and based on the results of a similar study by Esteghamati et al. [10], who reported a mean difference of 12.5 points in psychosocial adaptation scores (SD ≈ 9.5) between their intervention and control groups, the minimum required sample size for each group was estimated at 30 participants. Considering a 10% dropout rate, the final sample size was set at 40 patients per group.

Participants were selected from hospitalized patients with type 2 diabetes in the target hospitals who met the inclusion criteria. The inclusion criteria were as follows: diagnosis of type 2 diabetes for at least one year, age between 20 and 65 years, willingness to participate in the study, no prior participation in training programs related to psychosocial adaptation, ability to speak Persian, absence of disabling comorbidities, and no previous participation in training programs related to the 5A self‐management model.

The exclusion criteria were as follows: unwillingness to continue participation, having an excellent psychosocial adaptation score according to the Derogatis Psychosocial Adjustment Questionnaire, occurrence of acute conditions during the study (e.g., cardiac or respiratory problems or diabetic coma), and missing more than three consecutive sessions.

### 2.3. Randomization and Intervention

After selection and obtaining written informed consent from all eligible participants across the five hospitals, participants were randomly assigned to either the intervention or control groups using a simple randomization method with a random number table. Stratified randomization by hospital was not performed; instead, all eligible participants were pooled before random assignment to ensure comparable group sizes. To ensure that the study findings were not biased by any single clinical setting, this pooling approach allowed for a balanced representation of patients from all five hospitals in both the intervention and control groups, thereby minimizing setting‐specific confounding factors. To maintain single blinding, patients were not informed of their group allocation.

The 5A intervention was delivered by the researchers (nursing experts trained in the 5A protocol) in six 60–90‐minute sessions over four weeks as follows:

• Session 1 (Inpatient—Days 1‐2): Step 1: Assess. The researcher conducted a semistructured interview to evaluate the patient’s current diabetes knowledge, health beliefs, and motivation for self‐management.
• Session 2 (Inpatient—Days 3‐4): Step 2: Advise. Patients received personalized information about the risks of their specific diabetic complications and the clear benefits of lifestyle changes and medication adherence.
• Session 3 (Outpatient—Week 2): Step 3: Agree. A collaborative session where the researcher and patient set 2‐3 realistic and measurable goals (e.g., daily walking or consistent glucose monitoring) based on the patient’s preferences.
• Session 4 (Outpatient—Week 3): Step 4: Assist. Focus on identifying individual barriers (e.g., financial issues and lack of family support) and developing a concrete action plan, including stress management techniques.
• Session 5 (Outpatient—Week 4): Step 4: Assist (Continued). Refinement of the action plan and providing psychological coping strategies to enhance psychosocial adaptation.
• Session 6 (Outpatient—Week 4): Step 5: Arrange. Establishing a follow‐up schedule (via phone or clinic) and providing a resource guide for community support and emergency contacts.

All sessions used a mix of face‐to‐face counseling, educational booklets, and visual aids (posters/charts) to ensure clarity. The intervention began during the patients’ hospitalization, where the first two sessions (focusing on Assessment and Advice) were conducted face‐to‐face in the ward. Following hospital discharge, the remaining four sessions (focusing on Agreeing on goals, Assisting with barriers, and Arranging follow‐ups) were conducted face‐to‐face during weekly outpatient visits at the diabetes clinics of the respective hospitals. This ensured continuity of care and the implementation of the 5A framework as patients transitioned from acute hospital care to home‐based self‐management. Sessions included education on diabetes, nutrition, physical activity, blood glucose control, medication adherence, and stress management.

To minimize the risk of treatment contamination between the groups, several measures were taken: educational sessions were conducted in a dedicated room, 5A‐based materials were provided exclusively to the intervention group with a request not to share them, and session timings were managed to reduce interaction between participants of different groups.

### 2.4. Data Collection Instruments

Data were collected using the following questionnaires:

1. Demographic Information Questionnaire: This included data such as age, gender, education level, marital status, occupation, duration of diabetes, and treatments received.
2. Diabetes Knowledge Questionnaire (DKQ): This questionnaire consists of 27 items and evaluates patients’ knowledge across six dimensions: general knowledge, hyperglycemia, diabetes complications, nutrition knowledge, physical activity knowledge, and insulin knowledge. Content validity of the questionnaire was confirmed with CVI = 0.79 and CVR = 0.8 [21].
3. Diabetes Self‐Management Questionnaire (DSMQ): This 27‐item instrument measures self‐management behaviors in four domains: glucose management, physical activity, dietary control, and healthcare use. Content validity was confirmed with CVI = 0.91, and reliability was supported by a Cronbach’s alpha coefficient of 0.84 [24].
4. Derogatis Psychosocial Adjustment Inventory (PAIS): This 45‐item questionnaire assesses psychosocial adaptation across seven domains: healthcare orientation, work environment, family environment, sexual relationships, extended family relationships, social environment, and psychological disorders. Items are scored on a 4‐point Likert scale (not at all, a little, somewhat, and completely) ranging from 0 to 3. The total score ranges from 0 to 135, with higher scores indicating poorer psychosocial adaptation [11]. In the present study, the Cronbach’s alpha coefficient for the PAIS was calculated to be 0.88, indicating excellent internal consistency.

### 2.5. Data Analysis

Data were analyzed using SPSS Version 20. Descriptive statistics (mean, standard deviation, frequency, and percentage) were used to summarize the data. Between‐group comparisons for quantitative variables were performed using the independent *t*‐test or Fisher’s exact test. The primary analysis for comparing postintervention psychosocial adaptation scores between the groups was the analysis of covariance (ANCOVA), which controlled for baseline scores. Paired *t*‐tests were used as supplementary analyses to assess within‐group changes (pre‐ and postintervention) for each group. To compare changes between the two groups while controlling for baseline scores, ANCOVA was applied. To clearly differentiate between the cognitive, behavioral, and psychosocial impacts of the 5A framework, each construct was analyzed as a distinct outcome. While these variables are interrelated, separate ANCOVA models were employed for each (DKQ, DSMQ, and PAIS) to ensure the specific effect of the intervention on each domain was captured independently. A significance level of 0.05 was considered for all statistical tests.

## 3. Results

In this study, 80 patients with type 2 diabetes participated (40 in the intervention group and 40 in the control group). The demographic characteristics of patients in the two groups are presented in Table 1.

**TABLE 1:** Demographic characteristics of patients in the intervention and control groups.

Variables	Intervention group (*n* = 40) (%)	Control group (*n* = 40) (%)	*p* value
Age (years)—mean ± SD	52.3 ± 8.7	51.8 ± 9.2	0.78

*Gender—n (%)*			
Female	22 (55)	20 (50)	0.65
Male	18 (45)	20 (50)	

*Education—n (%)*			
Below diploma	15 (37.5)	17 (42.5)	0.82
Diploma	16 (40)	15 (37.5)	
Above diploma	9 (22.5)	8 (20)	

*Marital status—n (%)*			
Married	32 (80)	34 (85)	0.56
Single	8 (20)	6 (15)	

Occupation—*n* (%)			
Employed	18 (45)	16 (40)	0.64
Retired	12 (30)	14 (35)	
Housewife	10 (25)	10 (25)	
Duration of diabetes (years)—mean ± SD	7.2 ± 4.3	6.9 ± 4.1	0.72

*Treatment received—n (%)*			
Oral medication	25 (62.5)	27 (67.5)	0.62
Insulin	8 (20)	7 (17.5)	
Combination	7 (17.5)	6 (15)	

*Note:* The above table shows that the two groups did not differ significantly in terms of age, gender, education, marital status, occupation, duration of diabetes, and treatments received and were considered homogeneous (*p* > 0.05).

Table 2 shows the scores of psychosocial adaptations, diabetes knowledge, and self‐management in the two groups before and after the intervention. Before the intervention, there were no significant differences between the two groups in terms of psychosocial adaptation, diabetes knowledge, and self‐management scores (*p* > 0.05). After the intervention, the scores of psychosocial adaptations have been reduced, and diabetes knowledge and self‐management improved significantly in the intervention group (*p* < 0.001), whereas these changes were not significant in the control group (*p* > 0.05).

**TABLE 2:** Comparison of psychosocial adaptation, diabetes knowledge, and self‐management scores between the two groups before and after the intervention.

Variable	Time	Intervention group (*n* = 40)	Control group (*n* = 40)	*p* value^∗^
Psychosocial adaptation	Before	76.9 ± 7.6	71.2 ± 7.8	0.86
	After	58.6 ± 7.9	72.4 ± 8.3	< 0.001
	*p* value^∗∗^	< 0.001	0.12	

Diabetes knowledge	Before	15.3 ± 3.2	15.1 ± 3.4	0.81
	After	21.7 ± 2.8	15.4 ± 3.1	< 0.001
	*p* value^∗∗^	< 0.001	0.23	

Diabetes self‐management	Before	62.5 ± 8.7	61.9 ± 8.9	0.79
	After	78.3 ± 7.2	62.1 ± 8.5	< 0.001
	*p* value^∗∗^	< 0.001	0.18	

^∗^Comparison between the two groups using the independent *t*‐test.

^∗∗^Comparison before and after the intervention within each group using the paired *t*‐test.

To definitively determine the effect of the intervention while accounting for any minor baseline differences, an ANCOVA was performed.

Table 3 presents the results of the ANCOVA for comparing psychosocial adaptation scores between the two groups after controlling for baseline scores. After adjusting for preintervention psychosocial adaptation scores, a significant difference was observed between the two groups (*F* = 42.36, *p* < 0.001). The effect size (eta squared) was 0.35, indicating that 35% of the variance in psychosocial adaptation scores after the intervention was attributable to the educational intervention.

**TABLE 3:** Results of analysis of covariance (ANCOVA) for comparing psychosocial adaptation scores between the two groups after controlling for baseline scores.

Source of variation	Sum of squares	Degrees of freedom	Mean square	*F*	*p* value	Effect size (eta squared)
Baseline score	125.36	1	125.36	18.47	< 0.001	0.19
Group	289.47	1	289.47	42.36	< 0.001	0.35
Error	524.18	77	6.81			
Total	939.01	79				

Table 4 presents the results of the comparison of different dimensions of psychosocial adaptation between the two groups after the intervention. Following the intervention, significant differences were observed in all dimensions of psychosocial adaptation—including healthcare orientation, work environment, family environment, sexual relationships, extended family relationships, social environment, and psychological disorders in favor of the intervention group (*p* < 0.05).

**TABLE 4:** Comparison of different dimensions of psychosocial adaptation between the two groups after the intervention.

Postintervention psychosocial adaptation	Intervention group (*n* = 40)	Control group (*n* = 40)	*p* value
Healthcare orientation	10.2 ± 2.1	7.8 ± 2.3	< 0.001
Work environment	8.7 ± 1.9	6.5 ± 1.8	< 0.001
Family environment	11.3 ± 2.4	8.9 ± 2.2	< 0.001
Sexual relationships	7.8 ± 1.7	6.2 ± 1.9	0.001 >
Extended family relationships	9.5 ± 2.0	7.3 ± 1.8	< 0.001
Social environment	8.9 ± 1.8	6.7 ± 1.7	< 0.001
Psychological disorders	16.2 ± 3.1	21.4 ± 3.5	< 0.001

## 4. Discussion

The findings indicate a significant reduction in the total PAIS scores in the intervention group, which signifies an improvement in psychosocial adaptation in patients with type 2 diabetes, confirming its potential as a practical nursing intervention for diabetes care. In contrast, the control group showed no changes in psychosocial adaptation after the intervention. The significant reduction observed in the intervention group underscores the program’s high effectiveness in clinical settings. These results are consistent with previous research, including studies by Sadeghi Golestan et al. [22], Heydari et al. [25], Haj Mohammadi et al. [23], and Keyvan et al. [26]). However, what distinguishes the present findings is the program’s applicability with a specific focus on psychosocial adaptation, a critical factor in diabetes management.

The success of the 5A self‐management program is driven by three interrelated mechanisms that nurses can apply in practice.

### 4.1. Enhanced Disease Knowledge and Coping

The significant increase in diabetes knowledge scores after the intervention indicates that structured education within the 5A framework enables patients to reframe their illness experience. Nurses can utilize the “Assess” and “Advise” steps of the 5A model to identify knowledge gaps and provide personalized coping strategies, supporting proactive disease management.

### 4.2. Strengthened Self‐Management Skills

The improvement in self‐management scores after the intervention shows that the 5A model equips patients with practical skills (e.g., medication adherence and blood glucose monitoring). Nurses should focus on the “Assist” and “Arrange” steps to develop personalized care plans and connect patients with community resources, which directly improves clinical outcomes.

### 4.3. Increased Self‐Efficacy and Reduced Psychological Distress

The reduction in anxiety and depression symptoms highlights the 5A program’s role in boosting patients’ confidence [27]. Nurses can apply the “Agree” step to collaboratively set achievable goals, reinforcing patients’ sense of control and mitigating psychological barriers to adaptation.

While the present study focused on psychosocial and behavioral outcomes and did not directly measure physiological parameters such as HbA1c or hospital readmission rates, improved psychosocial adaptation is widely recognized in the literature as a precursor to better clinical outcomes. Previous research suggests that patients with better adaptation are more likely to adhere to treatment regimens, which may indirectly lead to lower HbA1c levels and reduced risks of complications [10, 28]. Similarly, while we did not assess cost‐effectiveness directly, enhancing adaptation through the 5A program could potentially reduce long‐term healthcare resource utilization by empowering patients to manage their condition more effectively at home.

### 4.4. Addressing Discrepancies and Implementation Challenges

Although most studies support the 5A self‐management model, Zhang and Wang [8] reported limited effects on psychosocial adaptation in patients with ankylosing spondylitis. This discrepancy highlights the need for nurses to

• Customize interventions:
   Adjust the duration and intensity of the 5A program according to patients’ specific needs.
• Monitor demographic factors:
   Evaluate variables such as age, literacy, and social support that may influence outcomes, using the “Assess” step of the 5A model to tailor interventions.

### 4.5. Recommendations for Nursing Practice

• Adopt the 5A model as a standard tool:
   Integrate it into diabetes education programs, discharge planning, and chronic disease management protocols.
• Prioritize training:
   Offer workshops for nurses on 5A implementation, emphasizing motivational interviewing and goal‐setting techniques.

In conclusion, the 5A self‐management program provides nurses with an evidence‐based and adaptable strategy to improve psychosocial adaptation in patients with diabetes. By integrating this model into clinical practice, nurses can achieve meaningful improvements in patient outcomes, quality of life, and healthcare system efficiency.

### 4.6. Study Limitations

The limitations of the present study included a small sample size, which may limit the generalizability of the findings; a short follow‐up period, which does not allow assessment of the long‐term effects of the intervention; and the conduction of the study in five teaching hospitals, which may restrict the generalizability of the results to other healthcare settings. Furthermore, the study population consisted solely of hospitalized patients with type 2 diabetes; therefore, the findings may not be generalizable to patients in nonhospital or community settings. Future research is needed to evaluate the effectiveness of the 5A program in outpatient and community‐based populations. Furthermore, this study did not include clinical markers (e.g., HbA1c) or long‐term health utilization data (e.g., readmission rates), which limits our ability to draw direct conclusions about the clinical and economic impact of the 5A intervention.

## 5. Conclusion

The results of this study indicated that the 5A self‐management program can significantly improve psychosocial adaptation in patients with type 2 diabetes. By enhancing patients’ knowledge and self‐management skills, this program can contribute to better disease control and reduce complications. Therefore, it is recommended that this program be implemented within the healthcare system to improve psychosocial adaptation in patients with diabetes.

## 6. Recommendations

### 6.1. Clinical Recommendations

1. Implement the 5A self‐management program as part of standard care for patients with type 2 diabetes.
2. Train nurses and other healthcare providers on the 5A self‐management program and its implementation.
3. Design and conduct educational programs based on the 5A model for patients with diabetes in healthcare centers.

### 6.2. Educational Recommendations

1. Integrate the 5A self‐management program into diabetes‐related courses in nursing and midwifery schools.
2. Organize workshops for nursing students on the 5A self‐management program and its implementation.
3. Design and implement patient education programs based on the 5A model for individuals with diabetes.

### 6.3. Research Recommendations

1. Conduct studies with larger sample sizes and in different settings to improve the generalizability of the findings.
2. Conduct longitudinal studies to evaluate the long‐term effects of the 5A self‐management program on patients’ psychosocial adaptation.
3. Conduct studies comparing the effects of the 5A self‐management program with other self‐management programs on patients’ psychosocial adaptation.

## Author Contributions

Rafat Rezapour‐Nasrabad performed the measurements, performed the analysis, and drafted the manuscript. Sina Nasrollahi‐Nasrabad assisted the corresponding author in study design, conducted the data analysis, and revised the manuscript. Sohaila Dehno Khalaji was involved in planning and supervised the work. Seyedeh Narjes Mousavizadeh discussed the results and commented on the manuscript.

## Funding

No funding was received for this research.

## Ethics Statement

This study was conducted in accordance with the Declaration of Helsinki. Written informed consent was obtained from all participants before enrollment. Participants were informed about the study objectives, procedures, potential risks and benefits, voluntary nature of participation, and confidentiality of personal information. After study completion, the control group participants were offered the opportunity to receive the 5A self‐management program materials.

Also, this study was approved by the Ethics Committee of Shahid Beheshti University of Medical Sciences (IR.SBMU.PHARMACY.REC.1402.085).

## Conflicts of Interest

The authors declare no conflicts of interest.

## Data Availability

The data that support the findings of this study are available from the corresponding author upon reasonable request.
